# Tricuspid Regurgitation Associated with Implantable Cardiac Devices: A Double-Edged Sword

**DOI:** 10.3390/jcm13185543

**Published:** 2024-09-19

**Authors:** Ștefan Iliescu, Luminita Voroneanu, Alexandra Maria Covic, Dragos Viorel Scripcariu, Cristian Stătescu, Adrian C. Covic

**Affiliations:** 1 Institute of Cardiovascular Diseases “Prof. Dr. George I.M. Georgescu”, 700503 Iasi, Romania; stefiliescu@yahoo.fr (Ș.I.); covic.alexandra@yahoo.com (A.M.C.); cstatescu@gmail.com (C.S.); 2 Faculty of Medicine, University of Medicine and Pharmacy “Grigore T. Popa”, 700115 Iasi, Romania; accovic@gmail.com; 3 Nephrology Clinic, Dialysis, and Renal Transplant Center, “C.I. Parhon” University Hospital, 700503 Iasi, Romania; 4 Department of Surgical Sciences, University of Medicine and Pharmacy “Grigore T. Popa”, 700115 Iasi, Romania; dscripcariu@gmail.com

**Keywords:** tricuspid regurgitation after cardiac implantable devices, prognosis, management, mortality, heart failure hospitalization

## Abstract

The use of cardiac implantable electronic devices (CIEDs) has increased considerably, becoming a cornerstone of management for patients with brady- or tachyarrhythmia or for the prevention of sudden cardiac death. On the other hand, tricuspid regurgitation (TR) associated with CIEDs is progressively accepted as a serious clinical issue; the prognostic impact of TR is profound, as it is independently associated with increased mortality and a higher risk of heart failure hospitalization. Additionally, the management of established CIED-related TR continues to be challenging, with limited options for intervention once significant TR has developed. The balance between the lifesaving benefits of CIEDs and the risk of TR underlines the necessity for cautious patient selection and innovative approaches to device implantation and management. This review highlights the clinical importance, underlying mechanisms and challenges associated with lead-related tricuspid regurgitation in patients with CIEDs.

## 1. Introduction

Over the years, cardiac implantable electronic devices (CIEDs) have expanded their usefulness beyond their initial use for pacing in bradycardia to encompassing a range of cardiac conditions, including heart failure and the prevention of sudden cardiac death. More than 730,000 permanent pacemakers (PPMs) and 330,000 implantable cardioverter-defibrillators (ICDs) are implanted annually worldwide [1]. In Europe, more than 3.8 PPMs per million inhabitants, 2.2 ICDs and 1.8 cardiac resynchronization therapy (CRT) devices are implanted yearly [2]. These numbers are likely to increase as the population continues to grow older and cardiovascular comorbidities become more prevalent. 

Cardiac implantable electronic devices can be divided into three main groups. Permanent pacemakers (PPMs) are the only effective treatment for bradyarrhythmias, consisting of a subcutaneous pulse generator and at least one lead, which usually passes into the right ventricle. Implantable cardioverter defibrillators (ICDs) help prevent life threats and require thicker leads to be able to deliver higher-intensity shocks. Lastly, cardiac resynchronization therapy (CRT) coupled with a defibrillator (CRT-D) or pacing (CRT-P) function has been shown to improve outcomes in heart failure patients by re-establishing interventricular synchrony. To be able to achieve this, it includes three leads: in the right atrium, right ventricle and coronary sinus (for left-ventricle pacing). 

Tricuspid regurgitation (TR) following the implantation of CIEDs is becoming an increasingly recognized clinical concern. It can develop in a significant proportion of patients after CIED implantation, with studies showing incidence rates as high as 44% [3]. Additionally, TR associated with CIEDs is being gradually recognized as an important clinical condition related to an elevated risk of heart failure and mortality [4]. 

This narrative review aims to bridge the gap between the clear, unquestionable benefit of CIEDS and the long-term risk associated with tricuspid regurgitation, focusing on pathophysiology, prognosis and prevention. To achieve this, a broad and thorough search was conducted on PubMed, Scopus and Embase, from inception to July 2024, by two authors. Searching by hand for relevant articles was performed on reference lists from textbooks, articles and scientific proceedings. A combination of keywords and medical subject headings (MeSHs) were used in the search, such as “tricuspid regurgitation after CIED”, “pacing”, “cardiac implantable devices” or “defibrillator” AND “tricuspid regurgitation after CIED/prognosis” or “tricuspid regurgitation after CIED/mortality” OR “tricuspid regurgitation after CIED/epidemiology” OR “tricuspid regurgitation after CIED/risk factors” OR “tricuspid regurgitation after CIEDs” AND “chronic kidney disease” OR “atrial fibrillation” OR “diabetes”. Data extraction was performed independently by two authors using standardized data extraction forms. When more than one publication of a study was found, only the publication with the most complete data was included. The extracted data included identifiable information, study outcomes, details of the study protocol and demographic data.

This review includes all eligible studies, such as reviews, commentaries, editorials, retrospective clinical studies and prospective or randomized controlled trials, that have been published in English. On the other hand, unpublished studies and topics unrelated to the criteria were excluded. The included studies focused on adult patients, aged 18 years or older, with cardiac implantable electronic devices and who developed tricuspid regurgitation with at least one measure of epidemiology, prognosis and/or its association with heart failure and mortality (Table 1). 

## 2. Mechanisms

Tricuspid regurgitation following CIED implantation is mainly derived from several main underlying mechanisms: implantation-related, pacing-related or device-related. 

The main cause of acute valve dysfunction is leaflet impingement, described as mechanical interference with leaflet mobility determined by the ventricular lead [5]. In a case series that included patients who required surgical valvular repair for severe TR caused by CIED, 39% had leaflet impingement, with the second most common finding being lead adherence to the valve, present in 34% of patients. Leaflet perforation occurred in 17% of the cases, while lead entanglement in the subvalvular apparatus occurred in only 10% of the patients [6]. 

Right ventricular stimulation opposes the normal depolarization of the ventricles, causing the left bundle branch block pattern on the electrocardiogram. These electrical changes also translate into mechanical abnormalities defined as ventricular dyssynchrony. In time, abnormal depolarization and subsequent contraction are responsible for right ventricle dilatation, which results in inadequate valve coaptation. 

At last, the presence of the lead can lead to fibrosis and expose the patient to a higher risk of endocarditis; additionally, repeated embolization of thrombi from cardiac leads can cause pulmonary hypertension and tricuspid regurgitation due to right ventricular enlargement. Finally, when necessary, transvenous lead extraction poses high-risk complications, such as valve avulsion [2].

Given that tricuspid regurgitation is a relatively common echocardiographic finding, the distinction between CIED-associated TR and CIED-related TR is of utmost importance. In the first case, the presence of a CIED lead across the tricuspid valve can coexist with TR without a clear cause–effect relationship between the two, and the evolution of the valve disease is dependent on the usual risk factors. However, when TR appears or worsens after implantation (CIED-related tricuspid regurgitation), a causal effect could be assumed. Still, to establish causality in CIED-related TR, there must be proof of valve–lead interaction [2].

## 3. Epidemiological Data 

The incidence of TR after CIED implantation is not exactly established and has varied from 7.2% to 44.7% [3]. Reported incidences have ranged from 30.6% or even 38%, as reported by Höke et al., to the 21.2% found by Kim et al. or 13% found by Seo et al. [7,8,9]. The different definitions of significant TR and TR progression are the main reasons for the discrepancies in various studies. Some studies have evaluated all grades of tricuspid regurgitation, while others consider worsening TR as the progression to moderate or severe levels following CIED implantation.

The largest review to date, conducted in 2023, included more than 66,000 subjects and showed that patients who received device implantation (*n* = 1008) were significantly more predisposed to worsening tricuspid regurgitation compared with controls (OR 3.18; *p* < 0.01) [10]. Among 7777 patients, the pooled incidence of at least a 1-grade worsening of tricuspid regurgitation following device implantation was 24%. A recent meta-analysis and meta-regression of 52 studies (three RCTs, 18 prospective studies and 31 retrospective studies) with a combined total of 37,350 patients found a 24% incidence of a ≥1-grade intensification in CIED-associated tricuspid regurgitation [11]. The total estimated incidence for a ≥2 grade increase was 11% (95% CI: 6% to 15%; *p* < 0.001). Strikingly, patients with CIEDs had a higher risk of developing new or worsening TR in comparison with those without CIEDs (OR: 2.44; 95% CI: 1.58 to 3.77; *p* < 0.001). The risk of CIED-associated TR did not significantly differ between patients with PPMs and those with ICDs (OR: 0.70; 95% CI: 0.45 to 1.11; *p* = 0.175) [3].

## 4. Device Type and Lead Features

Studies have found varying relationships between lead type and the risk of TR. In a meta-analysis by Zhang et al., the global incidence of TR was greater with ICDs than PPMs (29.18% vs. 22.68%), presumably because of thicker and stiffer ICD leads, but the difference was not statistically significant [12]. A systematic review and meta-analysis by Alnaimat et al. also found no statistically significant difference between lead type and risk of TR (OR: 1.12; 95% CI: 0.57–2.18; *p* = 0.71) [10]. Interestingly, two studies that were included in this analysis, conducted by Leibowitz et al. [13] and Wiechecka et al. [14], examined patients during the second and seventh day post-implantation and found worsening tricuspid regurgitation rates of 11.4% and 15.4%, respectively. The rates reported in these two studies are lower than the ones outlined by studies with longer follow-up periods. These findings might suggest that worsening TR post-implantation could take several months to manifest.

Theoretically, leadless pacemakers (LPs) may decrease the frequency of TR by avoiding lead–leaflet interaction and impingement. However, the currently reported data are conflicting. After conducting echocardiographic evaluations on patients both before and after LP implantation, Beurskens et al. compared the results with those from age- and sex-matched controls who had dual-chamber (DDD) transvenous pacemakers. The findings revealed that 43% (*n* = 23) of patients experienced more severe TR at follow-up (12 ± 1 months) than at baseline [15]. Furthermore, increased MV regurgitation was observed in 38% of LP patients (*p* = 0.006). LP therapy unexpectedly resulted in an increase in TV dysfunction, similar to the changes observed in patients with DDD transvenous pacemakers, and negatively affected MV and biventricular function.

The mechanism is not completely understood but may involve valve impairment during implantation, mechanical stress of the device on the TV or its subvalvular apparatus or pacing-induced RV dyssynchrony. By contrast, Vaidya et al. reported data derived from ninety patients who underwent LP implantation after a median follow-up duration of 62 days; 19% of patients with transvenous pacemakers had worsening TR by two or more grades, while none of the patients who received LPs experienced significant changes in TR (*p* = 0.017) [16].

While historically, CIED-related TR has been considered a primary tricuspid regurgitation, its pathophysiological particularities call for it to be reclassified as a distinct category [17]. Lead interaction with the valvular leaflets has common mechanisms with primary causes of regurgitation, whereas chronic desynchrony with subsequent right-ventricle remodeling is similar to secondary TR. 

## 5. Prognostic Impact of Tricuspid Regurgitation

The main studies that have estimated the mortality risk associated with TR due to CIEDs are presented in Table 2. The study performed by Delling et al. underlined that the risk of developing TR is twice as high after PPM implantation, even after accounting for factors like age, cardiac comorbidities and common factors of secondary TR. PPM-related TR was associated with increased mortality over a median follow-up period of 1.6 years compared to patients with PPM who did not develop TR [18]. Offen et al. analyzed data from the National Echocardiography Database Australia (NEDA), consisting of 18,797 adults with CIEDs and 439,558 without, who underwent echocardiographic evaluations over a period of 20 years. That study highlights the following key findings: (1) at least moderate CIED-associated TR is highly prevalent (23.8%), occurring twice as often as in patients without devices; (2) it is linked to a 1.6- to 2.5-fold higher risk of all-cause mortality compared to those without TR, even after adjusting for factors such as age, sex, left-heart disease or atrial fibrillation, with a particularly significant impact on younger individuals; and (3) the grade of TR is more decisive than the cause of TR in determining outcomes, as the observed mortality was similar among the patients with TR regardless of whether a device lead was present or not [19]. There is an association between even mild CIED-associated TR and higher mortality rates, with aHR value of 1.11 [1.06 to 1.17] for mild TR, 1.62 [1.53 to 1.72] for moderate TR and 2.42 [2.25 to 2.61] for severe TR. 

In a recent meta-analysis including eight studies with a median follow-up period of 53 months, the pooled adjusted HR for all-cause mortality associated with significant TR post-CIED was 1.64 (1.40–1.90) (*p* < 0.001, *I*^2^ 30.28% and Egger’s test *p*-value = 0.088) [26]. In the first meta-analysis and meta-regression analysis, and thereby the largest study to date evaluating the true incidence and prognostic implications of CIED-associated TR, similar data were found [11]. That study revealed that CIED-associated TR was linked to a 52% increased risk of all-cause mortality (adjusted HR [aHR]: 1.52; 95% CI: 1.36 to 1.69; *p* ≤ 0.001). The risk was even higher (69%) for patients who had at least moderate CIED-associated TR (aHR: 1.69; 95% CI: 1.40 to 2.04; *p* ≤ 0.001) [11]. 

Additionally, CIED-related TR might determine the remodeling of the right heart and worsened RV function, with more frequent hospitalization for heart failure (see Table 3). In a single-center observational study with a relatively small sample size (*n* = 165), Kanawati et al. reported that patients who developed CIED-related TR presented a higher rate of heart failure hospitalizations (63.6%) compared with those that did not develop CIED-related TR (34.7% (*p* = 0.001)), with the results being confirmed over long-term follow-up [27]. Their secondary analysis highlighted that the risk of hospitalization for heart failure increases only after 12 months post-CIED implantation (HR: 1.99; *p* = 0.034). In 2014, Hoke et al. reported a significantly higher rate of a composite outcome of survival and/or heart failure-related events (HR = 1.641, *p* = 0.019) in patients with CIED-related TR [7]. The previously mentioned meta-regression analysis revealed similar results, indicating that CIED-associated TR was independently linked to an increased risk of both heart failure hospitalizations and a composite outcome of all-cause mortality and heart failure hospitalizations (aHR: 1.82; 95% CI: 1.19 to 2.78; *p* = 0.006 and aHR: 1.96; 95% CI: 1.33 to 2.87; *p* = 0.001, respectively) [11]—see Table 4.

## 6. Comorbidities and Tricuspid Regurgitation after CIEDs and Their Prognostic Impact

Recognizing the influence of comorbidities on TR after CIED implantation is crucial for assessing risk and managing patient outcomes. In this section, we explore how the presence of certain comorbidities can influence the development and progression of TR and evaluate their prognostic impact regarding mortality and heart failure hospitalization (HHF). Most of the studies that we analyzed focused on coronary artery disease, heart failure, hypertension, chronic obstructive pulmonary disease, atrial fibrillation, diabetes mellitus and chronic kidney disease.

In the analyzed studies, the incidence of coronary artery disease (CAD) varied from 3% to 70%. Delling et al. showed that permanent pacemaker recipients are more likely to have CAD (41% vs. 21%) [18], while Al-Bawardy noticed a higher mortality risk associated with a history of CAD [21]. The number of heart failure patients also has varied widely between studies, from 20% up to 100% in studies focused on CRTs. Chodor et al. included 40.6% patients with heart failure; however, they noticed that left-ventricular ejection fraction did not influence the survival rate in the TR progression group [3]. In contrast, Abu Sham’a noticed that there was no difference in mortality based on NYHA class or ejection fraction [20].

Similarly, hypertension is another comorbidity frequently taken into consideration. Although Delling et al. showed that hypertensive patients are more likely to need cardiac pacing, this was not significantly correlated with tricuspid regurgitation [18]. Moreover, it does not seem to influence either heart failure hospitalization or mortality [27].

Chronic obstructive pulmonary disease seemed to be less prevalent in the studied groups (4.7–15%). Kanawati et al. included 48 patients with lung disease. Their results show that it was associated with heart failure hospitalization only after 12 months of follow-up (HR 2.93; 1.51–5.69; *p* = 0.002) [27].

The available data regarding the influence of atrial fibrillation (AF) on tricuspid regurgitation are conflicting. While some studies recommend AF as an independent predictor of TR, with amplified incidence detected in patients with persistent AF, others describe no significant association. A retrospective analysis of 2533 patients who underwent PPM implantation showed that only AF (HR: 2.07; 95% CI: 1.27–4.09) and a history of open-heart surgery (HR: 3.34; 95% CI: 1.68–6.68) were predictors of lead-related TR [9]. Cho et al. studied predictors of moderate to severe TR in a population of 530 patients with CIEDs and with or without structural heart disease. Persistent AF was recognized as an independent predictor of tricuspid regurgitation, with the main incidence detected in both patient groups: 21.8% in those with structural heart disease and 18.6% in those without it [29]. Similarly, in their prospective study, Van De Heyning et al. found that only AF was associated with tricuspid regurgitation after cardiac device implantation after adjusting for the baseline TR grade [30]. However, a retrospective study on 1670 patients failed to show a significant association between AF and progressive tricuspid regurgitation or heart failure hospitalization [28]. A meta-analysis of 37 studies, adding up to 8144 patients, found that there was no significant statistical association between tricuspid regurgitation after PPM implantation and baseline AF, age, LVEF or baseline mild TR [12]. However, the authors did notice that the all-cause mortality after one year of follow-up was higher in the group that experienced progression of TR [12]. In their univariate analysis, Riesenhuber et al. failed to show an association between atrial fibrillation and mortality [24]. 

Diabetes mellitus (DM) also seems to be an important prognostic factor. In the retrospective study by Lee et al., DM was associated with hospitalization for heart failure in univariable analysis. However, in multivariable analysis, only age, renal impairment (>stage 3) and larger LVEDV were independently linked to HF hospitalization [28]. In another study, developed by Riesenhuber et al., lower survival was noticed at 10 years in women with diabetes compared with women without it (43.7% vs. 55.2%, *p* < 0.001) [31].

Chronic kidney disease (CKD) could be a notable comorbidity in patients with TR after CIEDs. Patients with CKD are at a higher risk of developing TR after CIED implantation. Moreover, CKD is associated with a higher mortality rate in these patients (see Table 5). In a retrospective cohort study, the progression of TR in 990 patients with and without pre-existing right ventricular dilatation undergoing pacemaker implantation was analyzed. CKD was an independent survival predictor in patients with TR after CIEDs (HR: 1.62; 95% CI: 1.25–2.11; *p* < 0.001); other predictors included lead-associated TR, mitral regurgitation, heart failure and age ≥ 80 years [24]. Comparable results were observed in a retrospective single-center cohort study involving 6362 patients with PPMs. That study showed that the rates of device and lead replacements were higher in patients with cardiovascular comorbidities, including coronary artery disease, heart failure, hypertension, valvular heart disease, prior stroke/TIA, atrial arrhythmias or CKD [31]. In a multivariate COX regression, CKD was independently associated with decreased 10-year survival (HR: 1.83; 95% CI: 1.53–2.19). Additionally, in a large cohort of 1670 patients (27.8% with CKD), Wei-Chieh Lee et al. reported that moderate to severe CKD (stages 3-5) was an independent predictor of HF hospitalization in multivariate analysis (HR: 1.865; 95% CI: 1.008–3.450; *p* = 0.047) [28]. 

## 7. Strategies to Minimize the Risk of Tricuspid Regurgitation

As the prime mechanism of lead-related tricuspid regurgitation is the presence of a lead across the valve, the first measures to prevent this issue are related to the procedure. There are three main ways to place an RV lead: the “direct crossing technique”, where the tip is advanced directly toward the apex; the “drop-down technique”, where the tip is advanced through the TV but curved toward the right ventricular outflow tract; and the “prolapsing technique”, where the lead is curved so that at first, only the body is inserted into the ventricle, and it is then followed by the tip. Of all three techniques, only the third may reduce damage to the tricuspid leaflets [5]. Furthermore, lead placement in the ventricle impacts TR in two ways. Firstly, non-apical pacing (leads fixed on the septum or on the RV outflow tract) results in a more physiological pacing and decreases dyssynchrony, which may counteract the secondary nature of lead-related TR. Second, non-apically placed leads affect the way in which the body of the wire interacts with the valvular leaflets [5].

Yu et al. showed the way that the placement of the lead influences tricuspid regurgitation. They found that leads passing through the middle of the valve were less likely to increase TR, followed by leads that had a commissural position, while leads impending on one of the leaflets were associated with the highest proportion of TR increase. What is more is that apical leads were more likely to impede on a leaflet compared to non-apical leads, which were more likely to be positioned in the middle of the valve [34]. Current implantation techniques using fluoroscopy do not allow for accurate visualization of the tricuspid valve and its interaction with the pacing lead. It was proposed that transesophageal echocardiography could be used to aid lead implantation. The PLACE pilot study recruited 21 patients who underwent TEE-assisted lead implantation (in addition to fluoroscopy) and compared the results to a historical control group of 103 patients. Lead placement in a commissure was possible in 20 patients. At discharge, none of the active-arm patients showed increases in TR severity, while in the control group, TR worsened by one grade in 13.6% of patients and by more than two grades in 6.8% [35].

With regards to tricuspid regurgitation, His bundle pacing has two main advantages. Firstly, depending on the site of the block, it may not be necessary to insert a lead across the TV, and second, pacing the conduction system directly minimizes interventricular dyssynchrony. The risk of developing new TR seems to be low, and more importantly, there might even be an improvement in pre-existing TR [36].

Currently, there is no established strategy to prevent or reduce the incidence of CIED-induced TR. Since the positioning of the lead contributes to the leak, it is advisable to place the lead at the center of the valve or in the commissural areas and ensure that it does not obstruct leaflet movement. Although echocardiographic guidance during CIED implantation has been suggested, its implementation is challenging, and lead positions can change over time. Performing a transthoracic echocardiogram immediately after implantation and regularly during follow-up may help detect CIED-induced TR early, before fibrosis develops. Exploring alternatives such as leadless pacemakers, coronary sinus leads, epicardial stimulation or subcutaneous ICDs could also be beneficial options.

**Figure 1:**
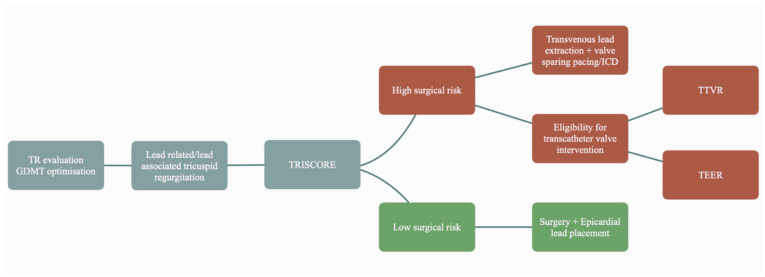
Management options in lead-related tricuspid regurgitation.

Another unanswered question is how to address patients with TR induced by a CIED (Figure 1). The first steps are evaluation of the severity of the tricuspid regurgitation and optimizing guideline-directed medical therapy. If the patient remains symptomatic, the next steps should be considered [37]. The most obvious answer would be to remove the lead. However, in reality, the problem is much more complex. The lead’s involvement in the mechanism of the regurgitation should be clearly demonstrated before transvenous lead extraction (TLE) [2]. Otherwise, this would render an already invasive procedure unsuccessful. An observational study on four cases showed little to no benefit of TLE in three of the patients. Furthermore, the authors showed that annular dilatation is linked to a worse response [38]. In a cohort of 119 patients with lead-related tricuspid dysfunction, followed for approximately 5 years after lead extraction, a study by Polewczyk showed a decrease in TR in only 35.29% of patients. These patients were shown to have better survival rates compared to the group with no significant change [39]. Additionally, this procedure carries risks such as damaging the subvalvular apparatus or the valve itself, which could worsen the leak, especially after a longer lead dwell time [2]. 

Beyond transvenous lead extraction, further management should be according to the patient’s individual characteristics. Surgical risk should be calculated using validated scores (TRISCORE [40]). In patients with low surgical risk, and especially in cases in which there is a mixed etiology of the leak, surgery is advisable, as it seems that lead-related tricuspid regurgitation has better outcomes compared to the other etiologies [41]. Furthermore, the mere presence of the lead adds complexity to the procedure, as separation is sometimes needed by blunt dissection. This might lead to damage of the leaflet requiring reconstruction [2]. 

In cases where there is a high surgical risk, TLE should be considered alongside with minimally invasive interventions. Transcatheter edge-to-edge repair (TEER) can only be performed in selected patients, and while transcatheter tricuspid valve replacement (TTVR) holds promise, there is currently limited clinical experience with this approach [42].

## 8. Conclusions

While lead-related tricuspid regurgitation and its pathophysiological cascade cast a grim shadow on prognosis, the benefits of cardiac implantable electronic devices are indisputable. Pacemakers remain the only effective treatment tool for bradyarrhythmias, while CRTs and ICDs significantly improve mortality rates. When there are a correct clinical indication and an accurate patient selection for CIEDs, and after all measures to minimize the impact have been taken, tricuspid regurgitation could be considered a necessary evil. 

## Figures and Tables

**Table 1:** Overview of studies on tricuspid regurgitation after CIED implantation.

Type of Study	Number of Studies	Total Sample Size	Follow-Up Duration	Tricuspid Regurgitation Incidence
Retrospective Clinical Trials	26	10,401 pts	5 mo–138.2 mo	10–39%
Prospective Clinical Trials	39	3936 pts	1.2 days–48.96 mo	1.3–41.2%
Randomized Clinical Trials	3	403 pts	12 mo–24 mo	6–23%
Device Types Used in these Studies				
Type of Device		Number of Studies	Total Sample Size	
PPM (Permanent Pacemaker)		37	7423	
LLPPM (Leadless Permanent Pacemaker)		7	314	
ICD (Implantable Cardioverter Defibrillator)		22	2587	
CRT (Cardiac Resynchronization Therapy)		16	2533	

**Abbreviations**: LLPPM: leadless permanent pacemaker; ICD: implantable cardioverter defibrillator; CRT: cardiac resynchronization therapy (with biventricular pacing leads); PPM: permanent pacemaker.

**Table 2:** Mortality risk by study.

Study	Sample Size	Study Design	Follow-Up (Years)	HR Death (95% CI)
Abu Sham’a et al., 2013 [20]	193	Prospective	2.2	6.7 (1.8–24.5)
Al-bawardy et al., 2015 [21]	1596	Retrospective	0.8	1.72
Delling et al., 2016 [18]	634	Retrospective	1.3	1.4 (1.04–2.11)
Grupper et al., 2015 [22]	689	Retrospective	3.3	1.36 (0.88–2.10)
Hoke et al., 2014 [7]	239	Retrospective	4.83	1.74 (1.00–3.03)
Papageorgiou et al., 2020 [23]	304	Retrospective	11.6	3.14 (1.29–7.63)
Riesenhuber et al., 2021 [24]	990	Retrospective	1.38	1.38 (1.04–1.84)
Stassen et al., 2022 [25]	852	Retrospective	7.6	1.74 (1.28–2.36)

**Table 3:** Hospitalization for heart failure (HHF) risk by study.

Study	Sample Size	Study Design	Follow-Up (Years)	HR HHF (95% CI)
Hoke et al., 2014 [7]	239	Retrospective	4.83	1.65 (1.04–2.5)
Seo, 2020 [9]	429	Retrospective	2.4	2.45 (1.43–4.22)

**Table 4:** Combined-outcome (death + HHF) risk by study.

Study	Sample Size	Study Design	Follow-Up (Years)	HR Death + HHF (95% CI)
Hoke et al., 2014 [7]	239	Retrospective	4.83	1.65 (1.04–2.5)
Seo, 2020 [9]	429	Retrospective	2.4	2.45 (1.43–4.22)
Lee, 2021 [28]	1075	Prospective	4.9	29.7% vs. 18.8%

**Table 5:** Impact of chronic kidney disease on survival outcomes.

Author Name	Sample Size	Study Design	Follow-Up	CKD Prevalence (%)	HR Death (95% CI)
Lee JW et al., 2010 [32]	870	Retrospective	4.9 ± 2.9 years	19.8%	2.63 (1.79–3.85)
Cho et al., 2019 [29]	530	Retrospective	7.6 years	54 (10.6%)	2.8 (1.54–5.10)
Marincheva et al., 2018 [33]	111	Prospective	6 months	30 (27%)	2 points
Riesenhuber et al., 2021 [24]	990	Retrospective	1.38 years	278 (27.8%)	1.62 (1.25–2.11)
Riesenhuber et al., 2020 [31]	6362	Retrospective	10 years	1069 (16.8%)	1.83 (1.53–2.19)

## Data Availability

The data presented in this study are available on request from the corresponding author.
